# Uncertainty assessment of proarrhythmia predictions derived from multi-level in silico models

**DOI:** 10.1007/s00204-023-03557-6

**Published:** 2023-08-01

**Authors:** Karolina Kopańska, Pablo Rodríguez-Belenguer, Jordi Llopis-Lorente, Beatriz Trenor, Javier Saiz, Manuel Pastor

**Affiliations:** 1 Research Programme on Biomedical Informatics (GRIB), Department of Medicine and Life Sciences, Universitat Pompeu Fabra, Hospital del Mar Research Institute, Barcelona, Spain; 2 Department of Pharmacy and Pharmaceutical Technology and Parasitology, Universitat de València, Valencia, Spain; 3 Centro de Investigación e Innovación en Bioingeniería (Ci2B), Universitat Politècnica de València, Valencia, Spain

**Keywords:** In silico toxicology, Drug-induced ventricular arrhythmia, Machine learning, Uncertainty, Variability

## Abstract

In silico methods can be used for an early assessment of arrhythmogenic properties of drug candidates. However, their use for decision-making is conditioned by the possibility to estimate the predictions’ uncertainty. This work describes our efforts to develop uncertainty quantification methods for the predictions produced by multi-level proarrhythmia models. In silico models used in this field usually start with experimental or predicted IC_50_ values that describe drug-induced ion channel blockade. Using such inputs, an electrophysiological model computes how the ion channel inhibition, exerted by a drug in a certain concentration, translates to an altered shape and duration of the action potential in cardiac cells, which can be represented as arrhythmogenic risk biomarkers such as the APD_90_. Using this framework, we identify the main sources of aleatory and epistemic uncertainties and propose a method based on probabilistic simulations that replaces single-point estimates predicted using multiple input values, including the IC_50_s and the electrophysiological parameters, by distributions of values. Two selected variability types associated with these inputs are then propagated through the multi-level model to estimate their impact on the uncertainty levels in the output, expressed by means of intervals. The proposed approach yields single predictions of arrhythmogenic risk biomarkers together with value intervals, providing a more comprehensive and realistic description of drug effects on a human population. The methodology was tested by predicting arrhythmogenic biomarkers on a series of twelve well-characterised marketed drugs, belonging to different arrhythmogenic risk classes.

**Supplementary Information:**

The online version contains supplementary material available at 10.1007/s00204-023-03557-6.

## Introduction

Ventricular arrhythmias, especially polymorphic ventricular tachycardia known as Torsade de Pointes (TdP), are very serious and feared adverse drug effects. The early estimation of the potential of drug candidates to induce ventricular arrhythmias is therefore of the highest interest to all stakeholders in healthcare (Gintant et al. 2016). The main mechanism of drug-induced ventricular arrhythmia involves the inhibition of one or multiple ion channels present in the membrane of ventricular myocytes. Such inhibitory effects prolong the action potential (AP) duration of ventricular cells triggering effects at the organ level. These prolongation effects can be observed at the patient level as changes in the duration and shape of QT-intervals on the surface ECG (Roden 2004; Yap and Camm 2003).

Since 2005, proarrhythmia assessment of pharmaceuticals for human use has been carried out according to the guidelines ICH S7b and ICH E14. In the non-clinical phase (ICH s7b 2005), the risk is estimated by combining results from in vitro inhibition assays of the rapid delayed rectifier potassium current (*I*_Kr_) encoded by the human ether-a-go-go-related gene (hERG) and an in vivo animal QT-prolongation studies, while in clinical phases (ICH E14 2005), drug proarrhythmia is assessed by measuring in vivo human QT/QTc interval prolongation. A decade later, the comprehensive in vitro proarrhythmia assay (CiPA) initiative enriched the mechanistic description of proarrhythmia and complemented the assessment by incorporating in silico methodologies (Fermini et al. 2016; Sager et al. 2014). The four-stage CiPA paradigm highlights the value of considering drug effects on a set of ion currents (*I*_Na_, *I*_NaL_, *I*_Kr_, *I*_to_, *I*_CaL_, *I*_K1_, and *I*_Ks_) as independent factors involved in arrhythmogenesis, instead of relying on *I*_Kr_, only (Li et al. 2017; Sager et al. 2014). The potency of drug-mediated inhibition of those ion channels, usually measured as the half-maximal inhibitory concentration (IC_50_), serves as input for electrophysiological models, which translate this information into proarrhythmia biomarkers (Li et al. 2019a, b; Park et al. 2019).

In the last decade, several efforts have been undertaken to enhance the assessment of proarrhythmia by introducing meta-models. Such meta-models are trained using larger series of simulation results, which allows for instantaneous predictions of selected proarrhythmia biomarkers. In particular, Mirams et al. (2014) described a meta-model built from simulated APD data for a series of different combinations variating the level of ion channel inhibition between 0 and 100% for five ionic transporters, including hERG, CaV1.2, NaV1.5, KCNQ1/MinK, and Kv4.3/KChIP2.2. Moreover, our groups also developed a multi-level in silico tool for the prediction of drug-induced action potential duration at 90% of repolarization (APD_90_) and QT-interval prolongation (Obiol-Pardo et al. 2011; Lucia Romero et al. 2018). The core of this tool was a large 3D data array containing a large number of simulated APD_90_ prolongation effects generated by the inhibition of three relevant ion channels (*I*_Kr_, *I*_Ks_, and *I*_CaL_). Since these values were pre-computed for a wide range of inhibition values, the method can provide an instantaneous estimate of the APD_90_ duration in ventricular cardiomyocytes, using as inputs the values of IC_50_s for these channels, and the plasma concentration of the drug. In a recent work, this approach was optimised by replacing the 3D data array with a Machine Learning (ML) model trained using only a small fraction of these costly computational simulations, leading to a significant reduction of the number of simulations required to obtain reliable APD_90_ estimates (Rodríguez-Belenguer et al. 2023).

Although computational approaches are a valuable complement to purely experimental methods, a detailed assessment of the variability and uncertainty associated with the predictions is required to increase the reliability of in silico methods (Gosling 2019). Quantification of variability and uncertainty in computational modelling systems and their predictions has been the objective of previous works in the cardiac safety field (Mirams et al. 2016; Mirams et al. 2020).

Several different methodologies have been described for the characterisation of variability observed when in vitro experiments are conducted to measure ion channel blockade produced by chemicals. Mirams et al. (2014) described the use of a meta-model for the characterisation of uncertainty in ion channel block and to further propagate these uncertainties considering a combination of channels. Chang et al. (2017) analysed the uncertainty and variability in drug-binding and drug ionic current block for TdP-risk assessment using the non-parametric bootstrap method and a Bayesian inference approach. Elkins et al. (2013) assessed the amount of between-experiment variability in drug-blockade of *I*_Kr_ (hERG), *I*_Na_ (NaV1.5), *I*_CaL_ (CaV1.2), *I*_Ks_ (KCNQ1/3ink), and *I*_to_ (Kv4.3/KchIP2.2) channels using concentration-effect curves fitted for positive control compounds from high-throughput-screening experiments performed at Glaxo Smith Kline and Astra Zeneca. Kramer et al. (2020) performed an extensive analysis of variability in results obtained from automated patch-clamp measurements across analysis sites and experimental platforms, thereby pointing out the importance of following the principles of Good Laboratory Practice (GLP) to minimise variability.

Another important source of variability is inter-individual differences among patients receiving the same drug treatment. When applying in silico approaches, the electrophysiological models that integrate ion channel-specific IC_50_ into ventricular arrhythmia biomarkers make use of a large number of parameters that were adjusted to fit experimental results. However, humans are not physiologically identical, and no single electrophysiological model can produce results suitable for representing all patients, nor accurately explain the observed differences between patients (Wisniowska et al. 2017). Population-based approaches have been described as a useful strategy to consider the inter-individual variability in the parameters of in silico models. Britton et al. (2013) analysed the inter-subject variability by generating a population of cellular AP models, each of which exerted small differences in parameters. These models were consequently filtered following physiologically based criteria and using acceptance–rejection criteria, as shown by Llopis-Lorente et al. (2022). Such populations of models can serve for the estimation of variability in the responses of a human population. Another approach for the analysis of biological variability was proposed by Johnstone et al. (2016), who used Bayesian statistics to infer distributions of inputs and parameters, such as current maximal conductance. Pathmanathan et al. (2015) performed an extensive analysis of uncertainty in the steady-state inactivation of the fast sodium current using an individual-based statistical method, the non-linear mixed effects (NLME) modelling, to analyse voltage clamp data taken from a population of cells.

Once diverse sources of variability and uncertainty in model inputs and parameters are identified, Uncertainty Quantification (UQ) analysis should be conducted to characterise and quantify their impact on models’ final outcomes. When input uncertainties are expressed using probabilistic terms, UQ is typically performed by applying sampling-based techniques to propagate them through the model, generating a distribution of model outputs. Monte Carlo (MC) simulations and Latin Hypercube Sampling (LHS) are the most popular methods for sampling-based uncertainty propagation (Clayton et al. 2020), but the application of other propagation approaches has been reported. For example, Sobie (2009) used multivariate regression for the assessment of the impact of variabilities in channel conductance, time constants, and steady-state voltage offsets. In the second case study described by Johnstone et al. (2016), they demonstrated the use of the Gaussian Process (GP) emulator to assess the effects of the uncertainties in AP model parameters once they are propagated to the output (Johnstone et al. 2016). Lately, Hu et al. (2018) described the use of polynomial chaos for the propagation of uncertainties and global sensitivity analysis within a multi-level cardiac electrophysiology prediction framework. In most published works, the UQ was performed only on a subset of model parameters. Pathmanathan et al. (2019) followed a different approach, suggesting that simpler models with a robust and complete UQ may be more useful than complex models without a full UQ. They performed the UQ on a canine cardiac cell model, which was reduced to relatively few parameters to which they assigned input distributions, controlled by a user-dependent hyperparameter.

In this work, we extend our multi-level in silico proarrhythmia model by integrating a comprehensive analysis of uncertainty. We start by identifying all sources of aleatory and epistemic uncertainty typically present in cardiac safety models. Focusing exclusively on aleatory uncertainty, we then investigate which of the identified sources affect the inputs of our model. We develop methods for the characterisation and propagation of the selected uncertainty types through the model, using applicable approaches and simple simulation methods, respectively. These methods aim to provide a more realistic representation of proarrhythmia biomarker predictions and allow for studying the individual and combined effect of different aleatory uncertainty sources on proarrhythmia biomarker predictions.

## Methods

### Multi-level in silico proarrhythmia model

**Fig. 1:**
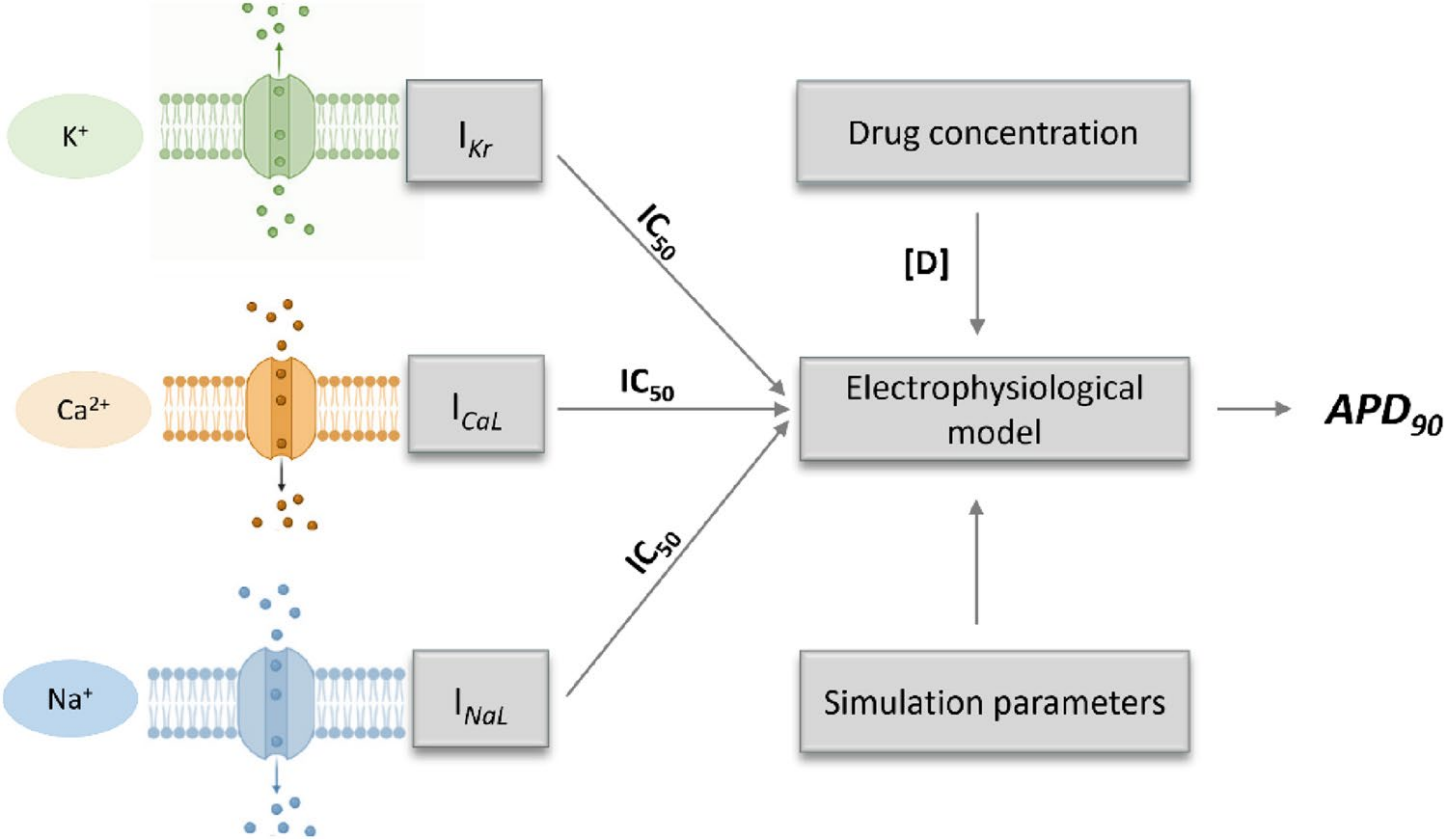
A simplified schema of our multi-level in silico proarrhythmia model. For a single compound, the input comprises a set of IC_50_ values for the currents *I*_Kr_, *I*_NaL_, *I*_CaL_, a drug concentration, and a set of electrophysiological simulation parameters. The model translates these inputs to an APD_90_ prediction

In 2011 and 2018, we published two works (Obiol-Pardo et al. 2011; Lucia Romero et al. 2018) describing the development and refinement of a multi-level in silico method for predicting cardiac safety biomarkers (APD_90_ and QT-interval duration). This prediction method, shown in Fig. 1, uses pre-computed simulations for estimating how compounds with different inhibitory effects on selected ionic currents can affect the ventricular tissue at certain concentrations. The inputs include IC_50_ values, obtained either in patch-clamp assays or predicted by in silico Quantitative Structure–Activity Relationship (QSAR) models, for three currents (here: *I*_Kr_, *I*_NaL_, *I*_CaL_), the drug concentration, and a set of electrophysiological simulation parameters. Recently, we developed an optimised version of this method in which the high number of pre-computed simulations was significantly reduced through the application of Machine Learning (Rodríguez-Belenguer et al. 2023).

### Electrophysiological simulations

In silico action potential (AP) modelling of the healthy human endocardial cardiomyocyte and APD_90_ measurements were done using the widely known model published by O’Hara et al. (2011), modified as described by Llopis-Lorente et al. (2020). Here, we considered drug effects on APD_90_ as a function of the three selected currents; *I*_Kr_, *I*_NaL_, and *I*_CaL_, which are considered particularly relevant for drug-induced occurrence of ventricular arrhythmias and are usually included in the pre-clinical ion channel screening panel at pharmaceutical companies (Chang et al. 2017).

### Electrophysiological Machine Learning model

We ran a set of electrophysiological simulations covering a wide range of combination of values for the ratio  ${\left(\frac{D}{{\mathrm{IC}}_{50,i}}\right)}^{h}$  for *I*_Kr_, *I*_NaL_, and *I*_CaL_. These ratios values were used to calculate channel inhibition via the simple pore block model (Eq. 1)

1 $${g}_{i, \mathrm{drug}}={g}_{i}{\left[1+{\left(\frac{D}{{\mathrm{IC}}_{50,i}}\right)}^{h}\right]}^{-1}$$

where *g*_*i*_, _drug_ represents the maximal conductance of channel *i* in the presence of the drug, *D* is the drug concentration, IC_50, i_ is the half-maximal inhibitory concentration for that drug, and channel *i* and *h* is the Hill coefficient.

The results obtained from the simulations (APD_90_) were stored in an array, consisting of three input values (IV) corresponding to *I*_Kr_, *I*_NaL_, and *I*_CaL_ channels. Each IV was calculated by taking the logarithm of the ratio ${\left(\frac{D}{{\mathrm{IC}}_{50,i}}\right)}^{h}$, as described in Eq. 2. For each channel (*I*_Kr_, *I*_NaL_, *I*_CaL_), the input value ranged from − 3 to 2.5, with a step increment of 0.1

2 $$\mathrm{IV} ={\mathrm{log}}_{10}\left({\left[\frac{D}{{\mathrm{IC}}_{50}}\right]}^{h}\right)$$

The standard utilisation of this array was as follows: for a given compound at a concentration D, Eq. 2 was applied independently for the three ionic channels (*I*_Kr_, *I*_NaL_, *I*_CaL_). The resulting values were rounded to the first decimal and constrained between − 3 and 2.5, i.e., if an input value was lower than − 3 or higher than 2.5, the value was then transformed to − 3 or 2.5, respectively. For each combination of the three calculated IV, the corresponding output (APD_90_) was retrieved from the array. For example, a drug with the following IC_50_s: 1 nM for *I*_Kr_, 1000 nM for *I*_NaL_, and 10 nM for *I*_CaL_ at a concentration of 1 nM yielded the data point [0, − 3, − 1], which led to an APD_90_ of 369.06 ms.

The results of these simulations (APD_90_) were used to build an SVM model, as described in Rodríguez-Belenguer et al. (2023). This model can be effectively used to predict APD_90_ for any compound with an *IV* within the range covered by the model training series. Indeed, this range expands from − 3 to 2.5 and is wide enough to represent the values found in most drugs and drug candidates. To limit the prediction space of this model, any *IV* minor than the minimum or superior to the maximum acceptable threshold is rounded accordingly. Hence, no values below − 3 or above 2.5 are used to predict the APD_90_ values.

### Uncertainty assessment protocol

According to EFSA’s “Guidance on Uncertainty Analysis in Scientific Assessments” (Benford et al. 2018a, b), UQ should commence with a comprehensive identification of all sources of uncertainty that have the potential to alter the assessment conclusion. In addition, the ECHA and the WHO recommend a complete and transparent characterisation of uncertainty in model inputs and the methodology by conducting a probabilistic analysis (European Chemicals Agency 2012; Organization and on Chemical Safety 2018).

In our protocol, the assessment question was defined as follows: “What is the APD_90_ that a certain drug will produce in an individual of a healthy population considering the compound’s potency of inhibition of the considered ion channels at a specific concentration?” As a first step, we identified all aleatory and epistemic factors that contribute to the uncertainty in the output used to answer the assessment question, when using the in silico proarrhythmia multi-level model. The next step was to investigate which sources of uncertainty affect the inputs of our model, thereby focusing specifically on the aleatory ones. Monte Carlo simulation was used to study how their effect on the input propagates through our model and is reflected on its output. Results of these simulations were expressed as values and intervals. The values can be interpreted as the most probable estimates of APD_90_ and the intervals as ranges of values within which the prediction could fall, given a certain level of credibility.

### Identification of the main sources of variability and uncertainty in cardiac safety models

**Fig. 2:**
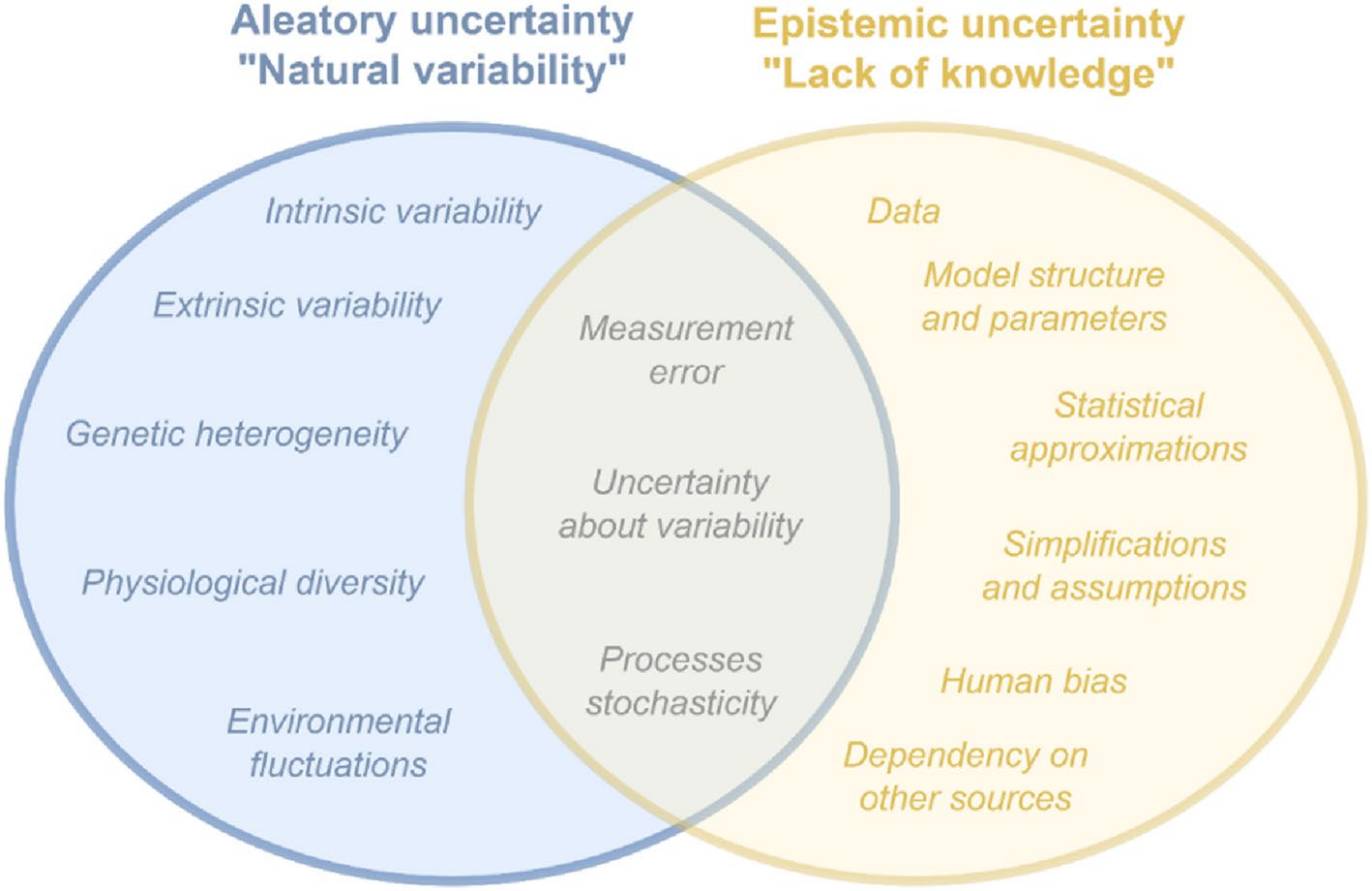
Identified sources of aleatory and epistemic uncertainty affecting elements of in silico multi-level proarrhythmia models

To correctly identify different sources of uncertainty, it is particularly important to distinguish between their aleatory or epistemic character (Benford et al. 2018a, b). To make a clear distinction, an overview of the most important sources of aleatory and epistemic uncertainties is presented in Fig. 2, adapted from Shamsi et al. (2020). The uncertainty types and the examples provided below apply to cardiac physiome models as previously described by Mirams et al. (2016).

The term aleatory uncertainty, which is used interchangeably with variability, refers to the indispensable heterogeneity and diversity that occurs within biological populations; let them be biological samples or human individuals. Variability, which can be controlled and measured but never completely removed, is reflected in multiple values that a quantity of interest can take on. Generally, variability can be subdivided based on the criteria, whether the differences are observed within the same subject (e.g., the same cell or the same person) or among different subjects (e.g., a collection of cells or a specific human population). These types are referred to as intrinsic or extrinsic variability, respectively. Aleatory uncertainty can also be classified considering the biological levels of organisation at which differences can be observed. Both, intrinsic and extrinsic variability can have their onset at the genetic (DNA of an organism), physiological (an organism), the environmental (population of organisms) levels, as well as at all intermediate levels that connect them.

On the contrary to aleatory uncertainty, when a parameter can only have a single true value but the knowledge to define it is lacking, it is described as epistemic uncertainty, shortly called uncertainty (Johnstone et al. 2016). In the context of computational modelling, epistemic uncertainty can be attributed to the model either through its inputs or through the underlying methodology. As for epistemic uncertainty in the inputs, it results mainly from incomplete data-gathering steps or the sparseness of the collected information. Concerning the methodology, uncertainty can have its origin in the structure of the model, in the selected algorithms and parameters or the introduced interpolation or extrapolation factors. The overall methodological process, including steps that proceed or succeed in the actual prediction, is also subject to epistemic uncertainty. These encompass all assumptions, simplifications, or statistical approximations made to develop the model or to interpret its results. Uncertainty can also arise as a result of coding errors or the failure to consider the dependency between sources of the required information.

Despite the theoretical differences, variability and uncertainty are tightly connected, since the epistemic uncertainty about a quantity of interest is often expressed based on a summary of aleatory uncertainty. More specifically, when the knowledge to define parameters for the characterisation of variability is generally incomplete, or the assumptions made to do so are incorrect, there is uncertainty about variability (Benford et al. 2018a, b). There are further cases when the separation between aleatory and epistemic uncertainty is not clear. A very well-known example is the occurrence of measurement errors that combine both the imprecision resulting from inevitable fluctuations in the measurement process and intrinsic and extrinsic variability between measurements of the same quantity (Johnstone et al. 2016).

### Sources of variability considered in this work

**Fig. 3:**
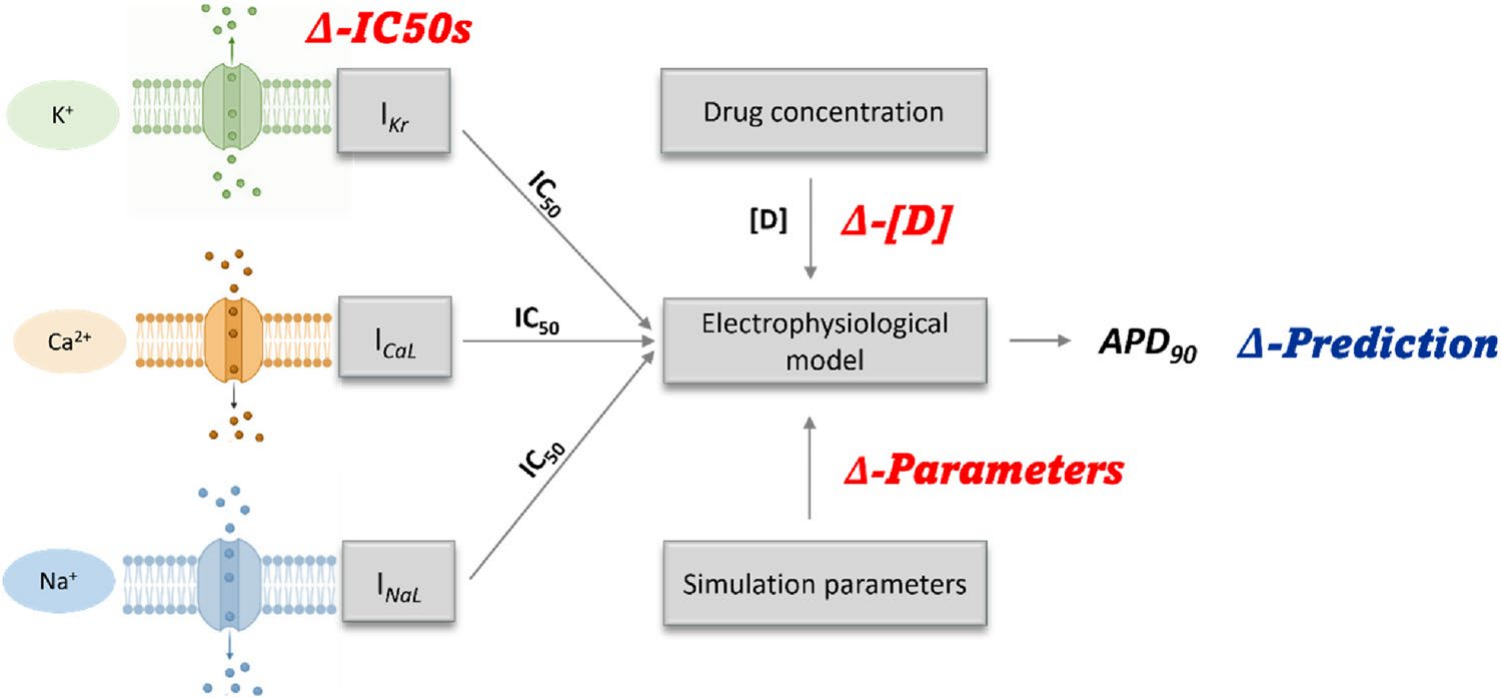
Structure of our multi-level APD_90_ prediction model showing the sources of variability that affect model inputs addressed in this work. *Δ-IC*_*50*_*s* represents the variability in the determined inhibitory drug effects on ion channels involved in physiological action potential generation. *Δ-Parameters* describes the variability in the electrophysiological model parameters due to inter-individual differences. *Δ-[D]* is the variability of the drug concentration obtained after the administration of the drug at therapeutic dosage due to inter-individual pharmacokinetic differences. Each of the input variability sources contributes to the overall level of output variability, indicated as (*Δ-Prediction*)

Computational models can simultaneously be affected by more than one source of uncertainty. In this work, aleatory uncertainty, which as mentioned above is mainly referred to as variability, was the only characterised and quantified subtype of uncertainty. Particularly, umbrella terms were used to group the variability sources that affect each specific input of our multi-level proarrhythmia model. The associations between model inputs and variability types were additionally marked within the basic structure of our model, as shown in Fig. 3. There are several epistemic factors associated with the inputs and the methodology, each of which can be reduced or even removed by filling the knowledge gaps. However, even if we acknowledge its importance, the quantification of epistemic uncertainty is out of the scope of this publication.

In our model, the inhibitory effects of drugs targeting ion channels are introduced as IC_50_ values. These values can either be measured experimentally or predicted using QSAR models for each considered ion channel. For IC_50_s measured experimentally, we assumed that the differences arising from intrinsic and extrinsic properties of analysed cellular systems can be summarised as *experimental variability (Δ-IC*_*50*_*s)*. Here, we also account for the imprecision of repeated laboratory experiments, since this factor cannot be separated from the measured values. Indeed, experimental variability could also be considered in the case of the Hill coefficient, which is a constant required to calculate the *IVs* for the model. Nevertheless, this constant is equal to one for many drugs, and even in a different case, the impact of a numeric change of *h* when computing *IV* (Eq. 2) is rather small (Parikh et al. 2017; Lucia Romero et al. 2018). Assuming that the consideration of one more source of variability with a minimal impact on the predictions could introduce additional complexity and potentially complicate the interpretation of results for those variability sources whose impact on the prediction outcome is more significant, experimental variability associated with the Hill coefficient was not considered in this work.

The second model input affected by variability is the parameters defined to conduct electrophysiological cellular simulations. Here, we talk about the *inter-individual variability* (*Δ-Parameters*) that refers to the differences between individuals in the population. To be more specific, in the context of this publication the umbrella term *inter-individual variability* unites practically all sources of aleatory uncertainty shown in Fig. 2. These include intrinsic and extrinsic differences between different cells within one human body and between several individuals, respectively. It also counts in genetic heterogeneity as well as environmental fluctuations, which together trigger different epigenetic modifications and, hence, physiological diversity between people and their hearts. Finally, when cardiac activity is measured experimentally, random and measurement errors may also be taken into account.

Another important model input affected by the presence of variability is the *drug concentration (Δ-[D])*. When assessing the arrhythmogenic properties of a compound, it is common to use the Effective Free Therapeutic Plasma Concentration (EFTPC) to describe the protein unbound drug concentration present in the blood of patients treated with therapeutic doses. However, the intrinsic and extrinsic variability also affects the pharmacokinetic (PK) processes of absorption, distribution, metabolism, and excretion, shortly ADME. Methods to address variability in drug concentration will be discussed later but will not be applied in our approach.

### Quantitative characterisation of selected types of variability

Different guidelines recommend to derive measures of variability from representative observation data containing multiple instances of the quantities of interest that follow a certain distribution of frequencies and their spread (Hastie et al. 2009; Shikano et al. 2012). Hence, the frequentist approach to probability was applied to characterise variability associated with the inputs of the multi-level proarrhythmia model. Incorporating pragmatic approximations based on different approaches described in detail below, it was assumed that experimental and inter-individual variability can be quantitatively described using normal probability distributions. The standard deviation (sd) was used to describe data spread.

#### Experimental variability in IC_50_

Variability in experimentally measured pIC_50_ (− log_10_(IC_50_)) was characterised by Elkins et al. (2013), who assumed that both the pIC_50_ and sd parameter are the same as, or very proximate to the one in control assays when the number of repeated measurements is high enough. The sd of the values measured in their study varied between ion channels, control compounds, and the number of repeats, reaching the minimum and maximum values of 0.08 and 0.2, respectively. Moreover, they showed that the pIC_50_ values collected in reiterated control assays on the same compound follow a logistic distribution.

We integrated these assumptions and represented the variability by considering that the experimental value is at the centre of a normal distribution, with an sd of 0.5. We chose a normal distribution for simplicity, due to its similarity with logistic distribution (similar in shape but with slightly higher kurtosis) (Hosmer Jr et al. 2013). The use of 0.5 is an approximation under the assumption that laboratory requirements stated in the GLP principles and stable testing conditions were not met during the measurement of IC_50_ values used in this work.

#### Inter-individual variability

To characterise the inter-individual variability, we applied the population-based approach previously described (Britton et al. 2013; Llopis-Lorente et al. 2022; Muszkiewicz et al. 2016; Sobie 2009)*.* A modified version of the widely used AP endocardial model developed by O’Hara et al. (2011) (O’Hara et al. 2011) was used as the baseline model. Assuming the baseline model represents the “averaged” model, an initial population of 1000 models was generated by randomly and simultaneously applying a scaling factor to the 15 channel conductances of the AP model. These scale factors modifying the channel conductances were randomly sampled from a normal distribution with mean 1 and standard deviation 0.2, and thus, assuring most of the population (> 99%) was in a range between ± 60% with respect to the baseline model. This range covers the natural variability reported experimentally in human ventricular tissues (Fink et al. 2008; Lucía Romero et al. 2009; Volders et al. 2000).

The 1,000 models were simulated at 37ºC and at the following extracellular concentrations: [Na^+^] = 140 nM, [Ca^2+^] = 1.8 nM and [K^+^] = 5.4 nM. Then, a calibration was performed. Plausible electrophysiological properties were defined according to experimental measurements for 15 biomarkers related to AP duration, amplitude of membrane potential, and calcium dynamics. Limits of acceptance for the 15 electrophysiological properties were taken from Table 1 in (Llopis-Lorente et al. 2022). These ranges were obtained from a variety of experiments conducted on different hearts and cardiac regions (Britton et al. 2017; Coppini et al. 2013; Grandi et al. 2010; O’Hara et al. 2011; Pieske et al. 2002; Sampedro 2020; Schmidt et al. 1998). After calibration, 860 models presented a plausible electrophysiological behavior according to experimental data. Sacling factors of the final population are available in “ORdmD scaling factors.xlsx” at https://riunet.upv.es/handle/10251/182593.

**Table 1:** Compounds belonging to the CiPA training and calibration set and their main characteristics including the EFTPC in nM, IC_50_ values in nM, the h, and the TdP and proarrhythmia risk class

Name	EFTPC (nM)	*I*_Kr_		*I*_NaL_		*I*_CaL_		Risk class
		IC_50_ (nM)	*h*	IC_50_ (nM)	*h*	IC_50_ (nM)	*h*	
Bepridil	33	144	1	339	1.9	638,000	4.6	High
Dofetilide	2	75	1	837,000	4.6	2,300,000	5.4	High
Quinidine	3,237	971	1	2,360	0.91	5,100,000	4.7	High
Sotalol	14,690	290,000	1	134,000,000	5.9	58,000,000	5.5	High
Chlorpromazine	38	650	1	673	1.8	6,350	2	Intermediate
Cisapride	2.6	72	1	421	2.2	4,050,000	5.6	Intermediate
Ondansetron	139	1,200	1	6,870	1.2	9,310,000	0.2	Intermediate
Terfenadine	4	129	1	98.3	1.1	1,220,000	5.2	Intermediate
Diltiazem	122	7,900	1	3,040	1.1	31,600	1.2	Low
Mexiletine	4,129	53,000	1	4,690	0.99	164,000	0.96	Low
Ranolazine	1,948.2	8,300	1	5,950	0.99	6,540,000	3.8	Low
Verapamil	81	460	1	982	1.2	11,200	0.8	Low

#### Population of input value combinations

The population of 860 models was used to generate a distribution of APD_90_ predictions for a given set of 125 input value combinations, selected to represent properties similar to those of real compounds. These values spread regularly along all dimensions in the 3D array covering all possible combinations (5^3^) of the five following values: − 3, − 1, − 0.5, 0, 1 for the three channels *I*_Kr_, *I*_NaL_, *I*_CaL_). Whether these distributions have the same shape and dispersion for diverse input values was first evaluated visually by plotting the value distributions as individual histograms.

**Fig. 4:**
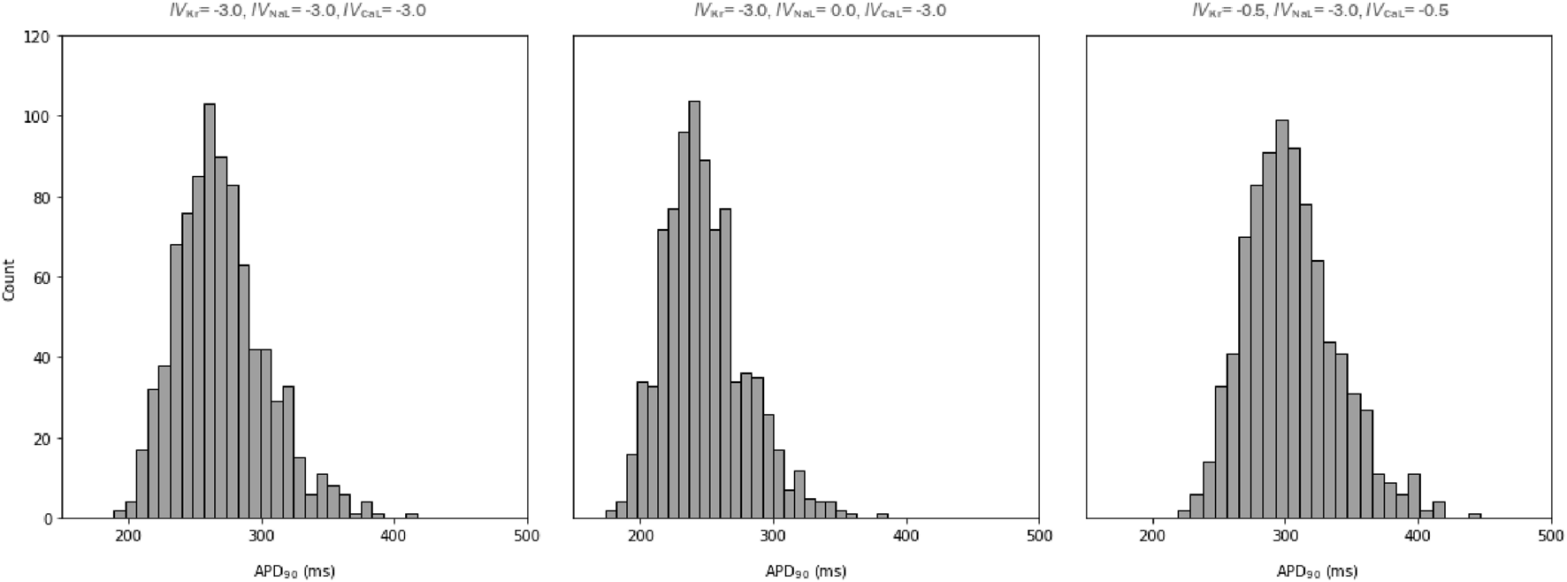
Distributions of APD_90_ values generated by the population of 860 electrophysiological models for input values #0 (left), #3 (middle), and #60 (right), from the input value combinations shown in the graphics

The example histograms in Fig. 4 represent the distribution of APD_90_ values obtained for three of these input value combinations. Left graphic, obtained using the input value combination (− 3, − 3, − 3), shows an APD_90_ distribution generated assuming no inhibition of the selected channels. The remaining two distributions illustrate distributions of output values produced for different input values combinations where inhibition was accounted for. The shape of the distributions is approximately normal (as checked using quantile–quantile plots) and for the 125 conditions tested, the average sd is of 35.4 ms, even if the dispersion is not homogeneous and different sd values were obtained for different input values.

The data table composed of 860 APD_90_ predictions generated for 125 input value combinations was used to build a model for predicting the dispersion (sd) of the distributions for a given set of input values. When generating predictions, this model produces an estimate of the dispersion of an APD_90_ distribution, for any drug with a combination of input values within the range covered by the models’ training series. This predicted dispersion can be seen as an approximation of variability associated with APD_90_ prediction due to the inter-individual differences in the electrophysiological parameters. The models were built using a method similar to the one described extensively in our previous work Rodríguez-Belenguer et al. (2023). SVM algorithm was used for the dispersion model, and the following hyperparameters were selected after optimising the model: C = 1, kernel = Radial Basis Function (RBF), gamma = Scale. The goodness of fit was assessed as per mean absolute error (MAE = 0.35) computed for the test set.

### Propagation and quantitative expression of variability in model outputs

Variability was propagated applying the forward Monte Carlo (MC) simulation approach (Kitagawa and Sato 2001). The MC technique belongs to a broader group of stochastic simulation methods that allow for the generation of random numbers to solve problems of non-deterministic nature. The advantage of such a method is that no assumptions about the model must be made. Moreover, the simplicity and simultaneous correctness of the methodology are very convenient. In the context of variability assessment, MC requires the identification of all random components of a model and defining their interactions with other elements. It is important to consider the correlation between the level of randomness, or variability, and the number of samples needed to propagate such variability, thereby maintaining the reliability of the result. In other words, the greater the spread parameter describing the variability, the more samples must be drawn from the probability distribution. Moreover, as the result is highly dependent on the assumed distribution to be sampled with the MC method, the preparatory work to make correct assumptions with regard to the random variables is particularly important (Kroese and Rubinstein 2012).

**Fig. 5:**
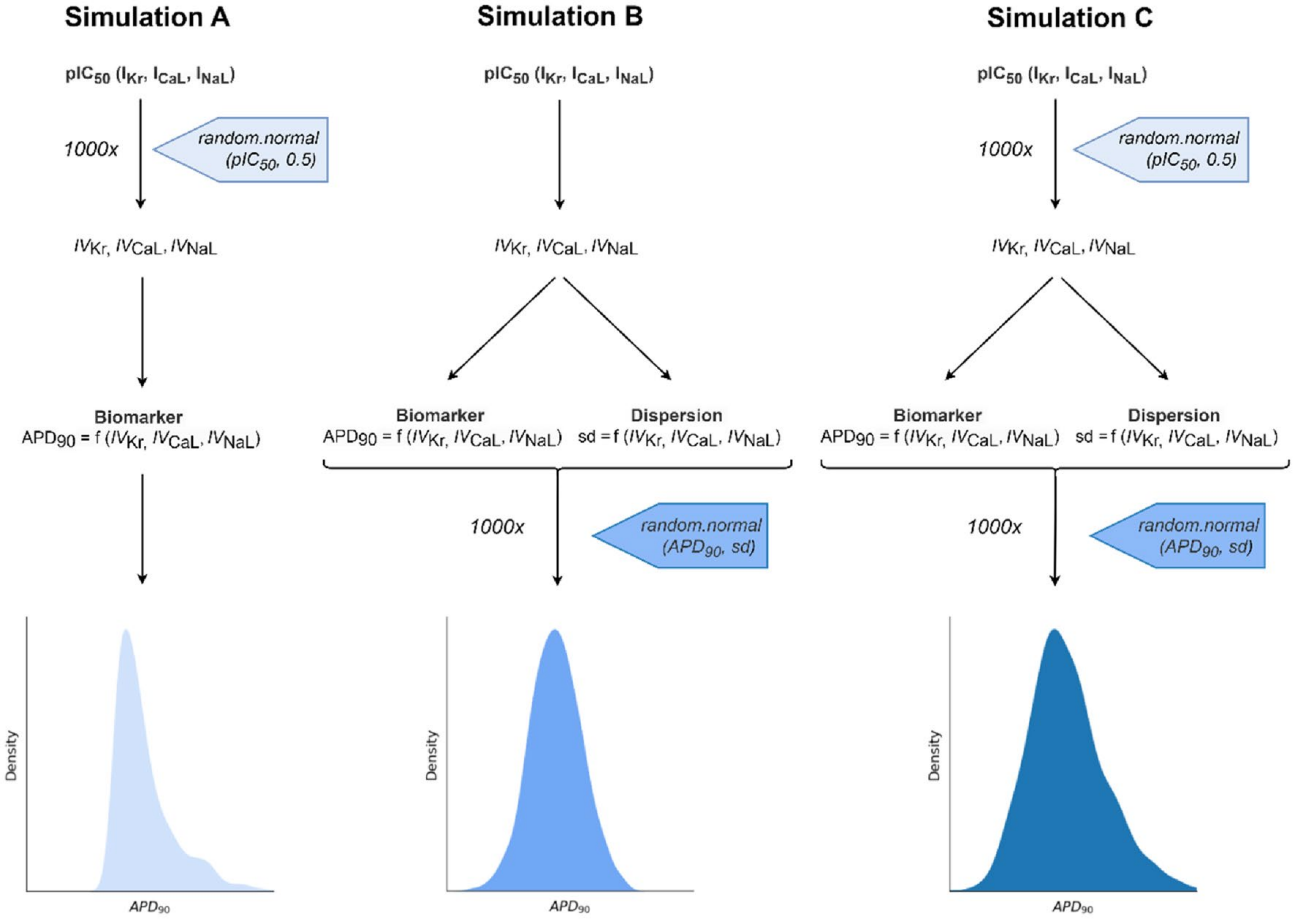
Schema of the three simulation types carried out in this work. Simulation A—propagation of experimental variability associated with pIC_50_ values, Simulation B—propagation of inter-individual variability arising at the level of electrophysiological model parameters, and Simulation C—propagation of combined experimental and inter-individual variability

The simulations were run considering only experimental variability (Simulation A), only the variability due to inter-individual differences (Simulation B), or a combination of both variability types (Simulation C), as shown in Fig. 5.

In all instances, the multi-level model described in Fig. 1 was applied 1000 times. In each simulation run, normally distributed random values were added up to specific elements of the model, using the random.normal(mu, sigma) function provided by the numpy library with a mu value of 0.0 and a sigma equal to the standard deviation of the variability represented, as described in the previous section.

In Simulation A, conducted to represent experimental variability in pIC_50_ values, the random value was added to the pIC_50_ used to compute the input values of the model. In Simulation B, aiming to represent the inter-individual variability, the model was run in the standard way, and once the prediction was generated, the random values were added to the APD_90_ results using the sd computed by the dispersion model. In either case, the procedure is equivalent to drawing the values from a normal distribution with the centre located in the original value and a standard deviation similar to the one obtained in the characterisation step. To analyse the combined effects of both types of variability, in Simulation C, both approaches were merged; prior to the application of the model, the pIC_50_ values were modified with the random values as in Simulation A and, after generating each APD_90_ prediction, the output values were modified by adding the random values as in Simulation B.

In all three cases, the simulations generate output distributions of slightly different APD_90_ values. The centre of these distributions (median or 50th percentile) was used as the point prediction, while the value range between the 10th and the 90th percentile was used as an interval representing the prediction variability, which can be interpreted as the 80% confidence interval.

### An example case study using CiPA compounds

To evaluate the practical application of our methodology, we applied it on a set of 12 CiPA compounds. These compounds, officially selected as the CiPA training and calibration set, were chosen in this study, because they belong to three risk classes (low, medium, and high) and are well characterised in terms of their arrhythmogenic mode of action. Moreover, these are real drugs, each of which inhibits the selected ion currents *I*_Kr_, *I*_NaL_, and *I*_CaL_ with a different potency at different therapeutic concentrations, resulting in a different combination of model input values. An overview of some important properties of the selected drugs extracted from Colatsky et al. (2016); Li et al. (2019a, b); Llopis-Lorente et al. (2020) is provided in Table 1.

To obtain biomarker predictions that correspond with the arrhythmogenic potential of the drugs in clinical practice, the *IV*s were calculated using experimental IC_50_ values for *I*_Kr_, *I*_NaL_, and *I*_CaL_ channels and the EFTPC. As the starting point, a single APD_90_ biomarker prediction was generated using our default model for each of the 12 compounds. Then, experimental variability and inter-individual variability were characterised for these compounds and propagated through the model using the three different simulation types described above (Fig. 5). For each drug, this procedure yielded a single biomarker prediction and an interval interpretable as an 80% confidence interval. These results were analysed in detail and critically discussed to evaluate the advantages of assessing the impact of input variability on the uncertainty in the output of the model, which contrasts with relying on single model predictions.

### Software

The electrophysiological simulations and the generation of the APD_90_ array were carried out using MATLAB version R2021b. These results are available online on the public repository of the Universitat Politècnica de València (https://riunet.upv.es/handle/10251/191820). The simulations were carried out using scripts written in Python 3.8. Machine learning models were built and evaluated using Scikit-learn version 0.24.2 (Pedregosa et al. 2011), NumPy version 1.19.5 (Harris et al. 2020), Pandas version 1.1.5 (McKinney 2010), and Statsmodels version 0.12.2 (Seabold and Perktold 2010). Graphics were generated with Matplotlib version 3.3.4 (Hunter 2007). All utilised data tables as well as the source code of the python scripts, models, and methods described here are freely accessible at GitHub (https://github.com/phi-grib/Cardiotox_uncertainty) and usable under GNU GPL v3 open source license.

## Results

### Overview

The main aim of this work was to develop methods for the assessment of uncertainty, mainly of aleatory type, in prediction results provided by the previously described in silico multi-level proarrhythmia model (Fig. 1). This model predicts the proarrhythmia biomarker APD_90_ of a certain compound from the experimentally measured or predicted inhibition potency of three ion currents (*I*_Kr_, *I*_NaL_, *I*_CaL_) for a given drug concentration and channel-specific Hill coefficient.

The protocol for uncertainty assessment and quantification involved three steps:

1. Identification of the main sources of variability and uncertainty in cardiac safety models
2. Quantitative characterisation of selected types of variability
3. Propagation and quantitative expression of variability in model outputs.

Independently of the type or source, we recognised that all uncertainty types identified (point 1) are interconnected and to some extent affect each other and the output of the model. Nevertheless, for this work, we attempted to group them based on their association with the model inputs. Later, we characterised and quantified the individual and the combined effect of two selected variability types (points 2 & 3) on the predictions generated by our model.

This method was applied to a set of 12 CiPA drugs. The results of this use case were analysed, considering the benefits that such output could provide for drug developers and decision-makers.

### Step 1: identification of the main sources of variability and uncertainty in cardiac safety models

Figure 2 presented in the section “Methods” provides an overview of the most important sources of aleatory and epistemic uncertainty generally associated with cardiac safety models.

The origin of aleatory uncertainty was identified as intrinsic and extrinsic variability, as well as measurement errors. These aleatory elements were used to find associations with the inputs of our model. As a result, we summarised them under the umbrella terms “experimental variability” and “inter-individual variability”, affecting the input IC_50_ values and the parameters predefined in the electrophysiological action potential simulations models, respectively. The experimental variability of the Hill coefficient required to compute the input values of our model was not considered in this work, due to its minor impact (see “Methods” section for details). Additionally, the drug concentration is also subject to aleatory uncertainty, mainly due to intrinsic and extrinsic heterogeneity among subjects of the same population, leading to differences in pharmacokinetic responses. Compared to the Hill coefficient, the impact of drug concentration on the numeric outcome of Eq. 2 computing the input values of the proarrhythmia model is larger. However, due to some limitations of this protocol, the impact of variability in drug concentration on the overall uncertainty levels in the prediction of the model was not quantified here.

With regard to epistemic uncertainty, the two main affected model components are the inputs from which the predictions are generated and the methodology underlying the prediction system. Experimental inputs are subject to epistemic uncertainty due to multiple unknown values and approximations introduced during laboratory measurements and in the consequent data processing. Some degree of epistemic uncertainty also accompanies all methodological steps, starting with the selection of models or algorithms, through the definition of their parameters and to the subjective expert judgements informing the model, to simplifications and assumptions accompanying the interpretation of the prediction results.

### Step 2: quantitative characterisation of selected types of variability

#### Experimental variability

Experimental variability was characterised based on assumptions and results previously published by Elkins et al. (2013). Here, we assumed that IC_50_ values measured for different cardiac ion currents and different compounds are naturally associated with levels of deviation of similar magnitude as those of control compounds in the published literature. In the simulations, this subtype of aleatory uncertainty was introduced by adding to the experimental pIC_50_ values a random value following a normal distribution with mean 0.0 and an sd of 0.5, as explained in the section “Methods”.

#### Inter-individual variability

Inter-individual variability was characterised following a multi-step approach based on a population of models. This model population, consisting of a total of 860 models, was generated by introducing variations into the default electrophysiological model used in this work as described in the section “Methods”. In particular, the parameters for every single model belonging to the population were equalled to those expected from a healthy population of patients. The population of models was then applied to predict APD_90_ values from a set of 125 input value combinations. The resulting 3D array served as training data to build a predictive model that can provide approximate estimates of the variability that can be attributed to the single APD_90_ prediction. This variability is expressed as predicted sd, as explained in the section “Methods”.

### Step 3: propagation and quantitative expression of variability in model outputs

The variability characterised in Step 2 was propagated through the model by running MC simulations, as shown in Methods in Fig. 5. The MC simulations conducted in this work incorporate only the experimental variability into the input values (Simulation A), add up inter-individual variability into the APD_90_ predictions (Simulation B) or combine both types of simulations (Simulation C). See the section “Methods” for details. In all instances, these simulations turned single inputs into a collection of 1000 differently distributed output values. These distributions can be seen as a means to complement single predictions provided by our initial model by an informative value interval. Being a product of each prediction, the centre of such interval corresponds to the centre of the APD90 distribution (median or percentile 50th) and ranges from the 10th to the 90th percentile. These intervals can informally be referred to as the 80% confidence intervals and represent the central range of values which the model would produce 80% of the times.

### An example case study using selected CiPA compounds

#### Value distributions resulting from variability propagation

To assess the practical value of the developed methodology, the above-described steps 1–3 were applied to a collection of 12 compounds with well-defined cardiac electrophysiology and proarrhythmia risk classes defining the severity of clinical effects, as previously characterised and published by the CiPA initiative (Colatsky et al. 2016). The use of these drugs was further justified in the “Methods” section.

**Fig. 6:**
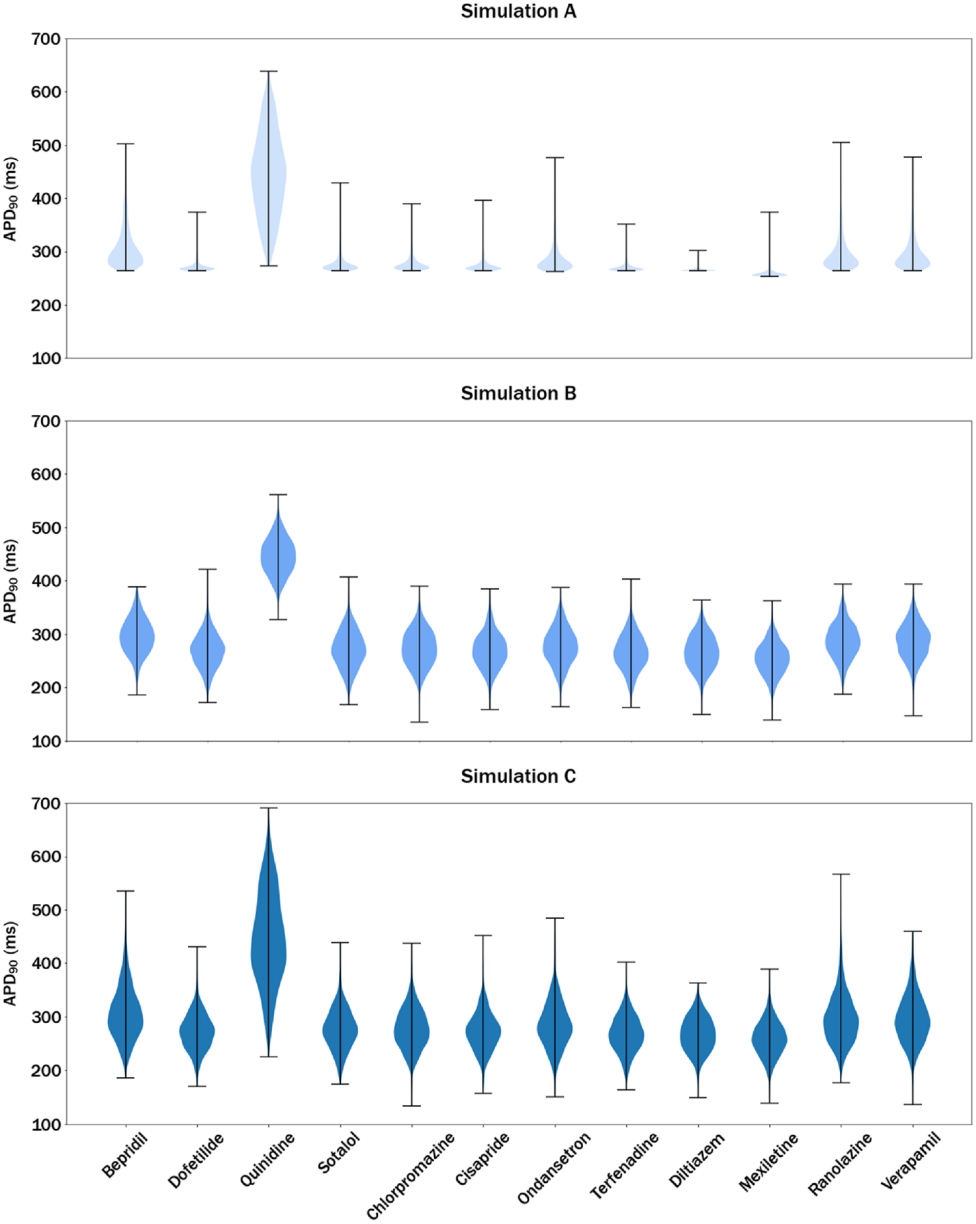
Violin plots showing distributions of APD_90_ values obtained in different runs of Monte Carlo simulations introducing the following variability types. Simulation A: Experimental variability (Δ-pIC_50_); Simulation B: Inter-individual variability (Δ-Parameters); Simulation C: Combination of experimental and inter-individual variability

Application of the methodology on the example of the CiPA compound set yielded a collection of 1000 APD_90_ values for each CiPA drug and the considered simulation type. Figure 6 shows three sections of violin plots, each representing results from the simulations A-C.

When comparing the distributions presented in Fig. 6, obtained by propagating experimental variability (Simulation A) with those where inter-individual variability was considered (Simulations B and C), there are remarkable differences in the width and skewness. As described in the section “Methods”, in Simulation A, random numbers were added to the pIC_50_ values used to generate the model *IV*s. Hence, the shape and width of the output distributions do not depend directly on the assumptions made to characterise this variability type.

Conversely, the dispersion and the form of the output distributions essentially depend on how sensitive the output values are to small *IV*s changes in a certain region of the training series space. To understand this concept, the model describing the non-linear relationship between the APD_90_s and the *IVs* can be visualised as a hyperplane. In some regions, this hyperplane is rather flat and, therefore, small changes on the *IV*s produce rather similar APD_90_ predictions. In other regions, this hyperplane is steeper wherefore small *IV* changes (e.g., due to a pIC_50_ increase for a highly relevant channel) produce significant APD_90_ variations. For the analysed drugs, most of the distributions generated in Simulation A are right skewed, with the maximum value far from the distribution centre. This can be explained by the non-linear relationship between the *IV*s and the APD_90_s: even if the *IV*s used in this simulation follow a normal distribution, the output values will not. Therefore, the propagation of experimental variability resulted in a condensation of APD_90_ predictions in a narrow area of around 275 ms and a great right skew of the distribution for the majority of the drugs included in this analysis. In the case of Bepridil, Ranolazine, and Verapamil introducing variability into the pIC_50_ values yielded *IV*s that fell within a sloped region of the prediction function, resulting in wider output distributions and minor right skew. The *IV*s computed for Quinidine, however, were spread differently producing a wide distribution of APD_90_ values with no notable skew.

As opposed to Simulation A, the dispersion and the form of distributions generated in Simulation B, shown in Fig. 6, are a consequence of the assumptions made about the inter-individual variability. Since they were generated by adding normally distributed random numbers to the output values, all APD_90_ distributions shown in Simulation B show a normal shape and exhibit no visible differences concerning the width. The minimal discrepancies in the width of the distributions can be justified with similarly minimal spread parameters predicted for these drugs as sd by the dispersion model (see the section “Methods”). As the minimum and maximum sd values in the training series of the dispersion model were 26.93 and 55.18 ms, respectively, these values marked the possible prediction range for any kind of input combination. But, since the *IV* combinations of the CiPA drugs did not reach these range limits, the predicted sd values to be considered as measures of the spread of each of these compounds varied between 31.64 and 37.21 ms. As this difference is quite a small relative to the predicted APD_90_ values, the observable differences between the width of the simulated distributions are minimal.

When combining both types of variability in one simulation run, we obtained the distributions shown in Fig. 6C. In general, they are rather similar to the ones obtained in Simulation B, but with a slightly larger dispersion and a little skew. Importantly, the effect of considering both kinds of variability simultaneously is not additive, and the effect depends on the drug studied. For example, these effects were particularly noticeable for Bepridil, Quinidine, Ondansetron, Ranolazine, and Verapamil.

When comparing all three approaches, an additional difference between the plots is the sudden cut-off observed for the results of simulation A, where only experimental variability was considered. This cut-off is absent in distributions resulting from Simulations B and C. This difference can be explained by the limited range of *IV*s used in the model describing their associations with the pre-computed APD_90_s (see the section “Methods”). This means that any variation of the *IV*s resulting in a decrease below the minimum value considered in the model (− 3.0) generally does not result in any change of the output. As a consequence, many of the 1000 *IV*s generated during the simulation were simply converted into the cut-off values and produced exactly the same APD_90_ output. For many drugs, this effect was observed for the *I*_CaL_ channel, the inhibition of which usually requires the drug to be administered at higher concentrations. In comparison, the inhibition of the *I*_Kr_ channel at the EFTPCs of the CiPA drugs is more common, due to which the *IV*s computed for hERG channel had the greatest impact on the predicted APD_90_. Conversely, the propagation of inter-individual variability in Simulation B added random numbers to the output values and was therefore not affected by these *IV* cut-offs.

In other words, in the case of Simulation A, after random values were added to the model inputs, these values were further processed by the model. In Simulation B, however, just one single value was predicted, and the distribution of values was simulated from the expected distribution parameters.

### Value intervals as a quantitative expression of uncertainty in the output

**Fig. 7:**
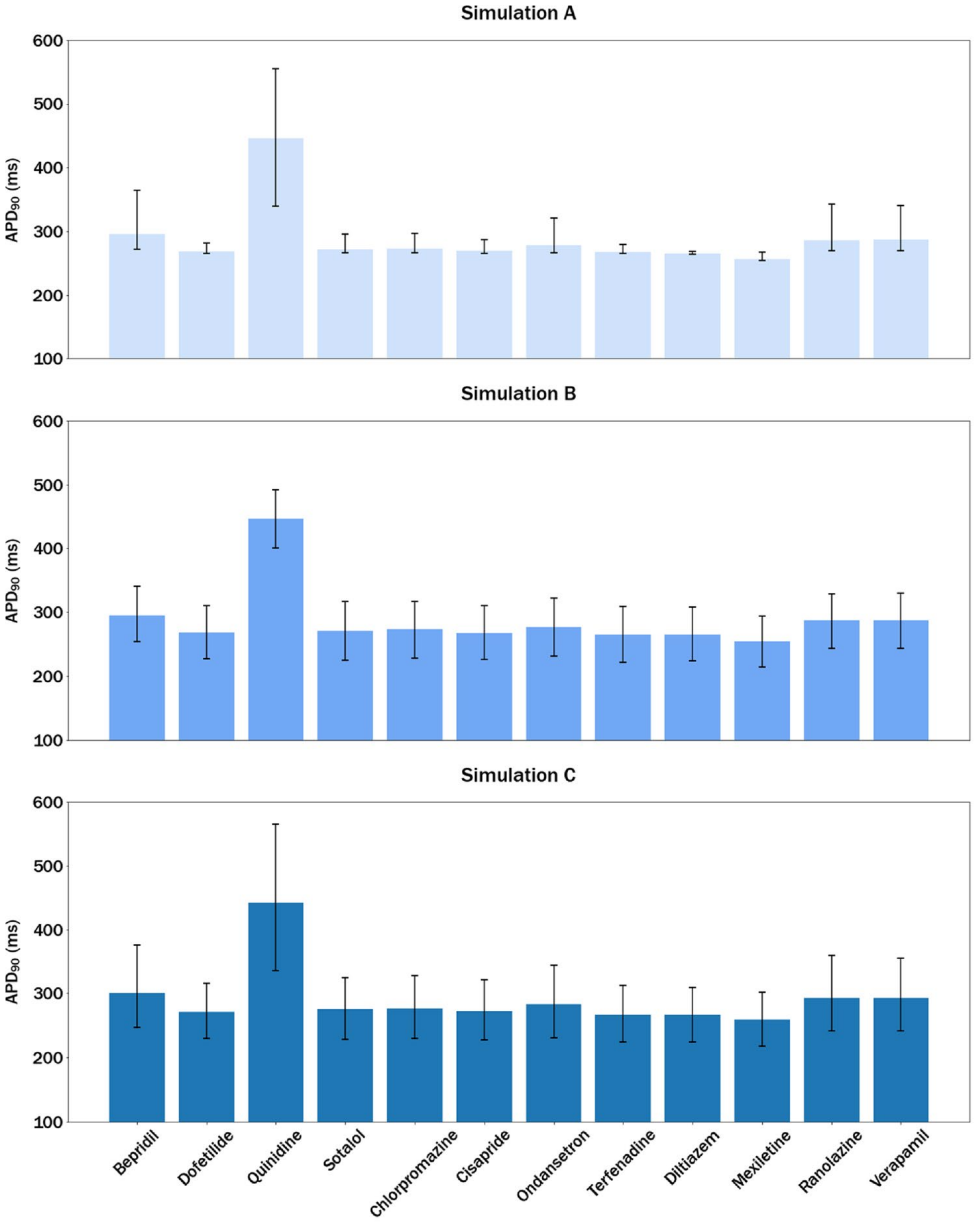
Bar plots showing the median of the APD_90_ predictions obtained for the 12 CIPA compounds, using three simulation types. Simulation A: Experimental variability (Δ-pIC_50_); Simulation B: Inter-individual variability (Δ-Parameters); Simulation C: Combination of experimental and inter-individual variability. The intervals represent the 10th and 90th percentiles obtained from the distributions shown in Fig. 6

The distributions of the predicted APD_90_ values were used to obtain intervals between the 10th and 90th percentiles for the 12 CiPA compounds, yielding the results shown in Fig. 7.

The bar plots in Fig. 7 show no remarkable differences in the APD_90_ predictions generated in three different simulations conducted for the same drug. This observation allows concluding that the actual prediction, computed as the median value of the APD_90_ distributions produced in simulation A–C, is barely affected by the simulation type and the biomarker prediction can be expected to remain unchanged. On the contrary, important differences can be observed in the widths of the intervals obtained by the different simulations. For Simulation A, these intervals vary between 4.3 and 216.6 ms, with a maximum difference exceeding 200 ms. In contrast, the intervals obtained for Simulation B are relatively similar for all tested compounds and range from 79.3 and 92.9 ms, approximately, thereby showing a maximum difference between two compounds of 13 ms. An overall increase in the intervals’ width is noticeable when combining both types of variability. However, combining two sources of variability does not lead to additive results, meaning that the combined result is not the sum of the two sources. Considering that the predicted numeric values could be potentially used to assign compounds into different risk classes (of TdP or arrythmia), it is possible that the interval ranges cross the boundaries of different classes, making then difficult to assign the compound to one of them.

**Fig. 8:**
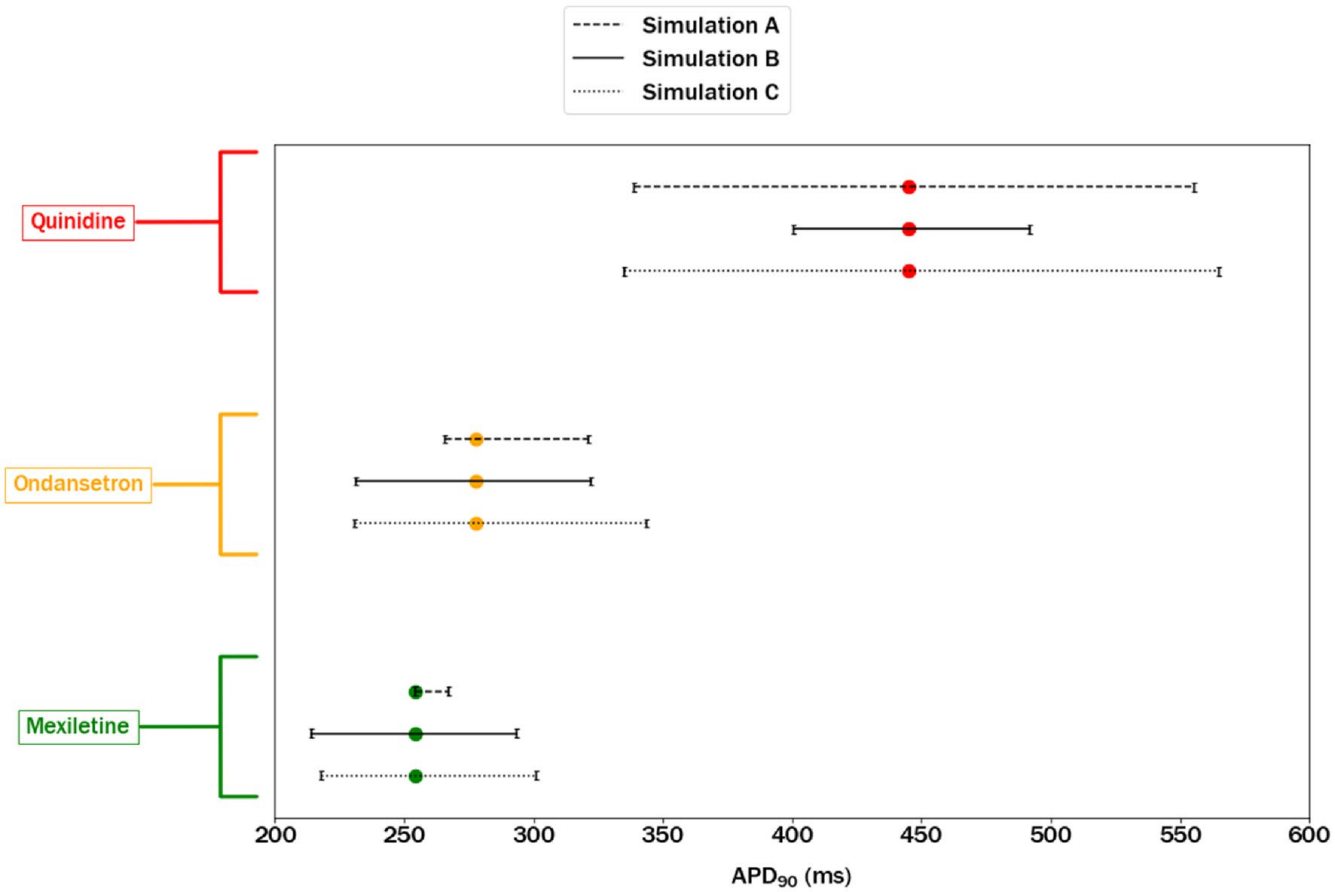
Predicted APD_90_ values and their corresponding 80% intervals for three selected CiPA compounds assigned to the following arrhythmogenic risk classes: Quinidine as a high-risk drug (red); Ondansetron as an intermediate-risk drug (orange); Mexiletine as a low-risk drug (green). Intervals shown here were obtained in MC Simulations A–C as described in Fig. 4. (color figure online)

To illustrate this situation, we have shown in Fig. 8 the prediction intervals for Quinidine, Ondansetron, and Mexiletine belonging to the high-, intermediate-, and low-risk class of TdP, respectively, as defined by the CiPA initiative (Colatsky et al. 2016).

It can be seen that the intervals computed for high-risk and low-risk drugs using any of the presented approaches do not overlap and would allow a clear class assignment. On the contrary, the APD_90_ interval computed for the intermediate-risk compound overlaps the interval of the low-risk class using all three simulation scenarios as well as the high-risk class when the most conservative scenario is used. In general, the use of APD_90_ predictions intervals, compared with appropriately selected critical values, would allow for a more conservative classification approach, which incorporates into the prediction both the effects of the experimental and inter-individual variability.

## Discussion

Obtaining a reliable risk evaluation for new drug candidates is one of the primary responsibilities of safety pharmacology. Regarding arrhythmogenic risk, the CiPA paradigm provided a standardised way for performing in vitro/in silico*-*based cardiac safety assessment using proarrhythmia models (Hwang et al. 2020). Despite the availability of a wide range of cardiac safety models stemming from the CiPA work, uncertainty analysis has been one of the last missing pieces to be addressed within this paradigm. Is in that context that this work proposes a protocol for the assessment of uncertainty and variability applicable to multi-level in silico proarrhythmia models.

### A critical view on the developed methodology

#### Experimental variability

The central hypothesis behind this work is that there is a “true” pIC_50_ value when one specific ion channel is exposed to a certain concentration of a drug in one specific moment in time. However, the notion of a “true” pIC_50_ is relatively idealistic and therefore does not correspond to what can be expected in practical situations. This is because in the proposed “Uncertainty assessment protocol”, the arrhythmogenic potential of drugs is assessed using a specific computational model and a combination of input values which are affected by multiple aleatory factors contributing to the overall levels of experimental variability. Hence, the consideration of experimental variability in cardiac safety model inputs is a step toward increased credibility of the predictions obtained from such models.

In this work, we assumed the same spread measure and the normality of the distributions describing the variability in the inhibition of each considered channel by each analysed drug. Even though the introduced assumptions were rather simple, they allowed to test the effect of this variability in the final prediction, at a proof of concept level. In practice, since each pharmaceutical company has individual methods to perform the inhibition tests the standard deviation considered could be adjusted to match the characteristics of the analytical platform, as well as the structure and properties of the tested compounds. Importantly, in this study, we considered the overall variability arising during the experiments, thereby combining the experimental errors with the biological properties of the samples. In the study performed by Lei et al. (2020), the authors demonstrated that the extent to which the artefacts in patch-clamp experiments affect the overall levels of experimental variability is much greater than the cell–cell or between-cell differences. Indeed, adding this additional layer of detail to separate experimental errors from intrinsic/extrinsic variability would contribute to a better understanding of the toxicodynamic effects of drugs in the context of cardiac safety assessment.

### Inter-individual variability

As for experimental variability, the consideration of inter-individual variability in cardiac model inputs can be seen as a step in the direction of realistic cardiac safety assessment. As described by Wisniowska et al. (2017), “Humans vary, so cardiac models should account for that too…”. The importance of considering inter-individual differences with regard to drug effects is particularly important if it comes to the protection of individuals who are more prone to develop cardiac arrhythmias or TdP. The use of a population of models to account for such differences allows to obtain different AP responses under the same pharmacological intervention. As compared to classical simulation methods based on an averaged model producing unique values, another advantage of populational approaches is that they provide novel insights into physiological and pathophysiological variabilities (Ni et al. 2018). In addition, this approach has shown that TdP-risk assessment improves when taking into account the electrophysiological variability between cells (Llopis-Lorente et al. 2022), therefore, increasing evidence points to the crucial role of variability in cardiac electrophysiological function.

Important to consider, however, are the characteristics of the population of interest. In this work, the electrophysiological model parameters, as well as the pre-processing of the simulated data, were based on criteria reflecting the attributes of a healthy population. Hence, to predict outcomes for a population with any type of underlying conditions, the first calibration step of the population of models would need to be modified accordingly to account for specific characteristics of this population.

It is worth noting that the described approach for representing inter-individual variability was based on the assumption that variability equally impacts all the 15 channel conductances and that this variability is independent for each parameter of the electrophysiological model. These assumptions were based on a series of results presented in the available literature on this topic. Nevertheless, further modifications of the proposed methodology allowing to assign unequal measures representing the variability in the conductances of different ion channels and to consider possible dependency between these measures could add additional value.

In the context of this work, however, establishing identifiability of the true ion channel conductance values was not the aim. For interested readers, different strategies for the identifiability of the parameters of the AP model are presented in the review by Whittaker and colleagues (Whittaker et al. 2020).

### Combination of variability

When combining experimental and inter-individual variability to produce a reasonable representation of proarrhythmia predictions, the emphasis should lie on appropriate interpretation of such results. From the theoretical perspective, the consideration of experimental variability is not necessary in clinical settings. Therefore, results obtained by combining these two variability sources do not intend to represent the variability in biomarker response that would be observed in a healthy human population. Nevertheless, when using computational proarrhythmia models which integrate some experimental values to produce estimates of human responses, the consideration of experimental variability is essential. In the latter case, the produced range of values aims to represent the variability in predictions, given the limited ability to define the “true” pIC_50_ values together with inter-individual differences among subjects of a population.

As shown in this work, combining variability, or other types of types or uncertainty, does not mean that the effects of each source on the final prediction will sum up. Nevertheless, as the current methodology for combining different variability types affects the shape of the obtained distributions, the methodology could be adjusted to account for this dependency. To do so, an additional analysis of the dependencies between each of the input sources, as well as of their associated variabilities, could be included in future work.

### Representation of results

Another important question is whether representing simulation results as a biomarker prediction with a corresponding 80% confidence interval has an advantage over standard methods yielding point estimates, only. As concluded by Sahlin (2015) “… a confidence interval is just an interval. It does not provide enough information to calculate an expected value or conservative value, which is important in rational decision making”. However, a confidence interval provided together with the expected value is very useful for communicating uncertain results in a simple way. Such intervals allow for the inspection of values that would be produced in experiments or for individuals that do not represent the exact centre of the distribution from which they were drawn. Since variability is an innate element of all-natural and investigational processes, assuming that a fixed prediction is the exact centre of a specific distribution is rather ingenious. However, when intervals are provided together with single values to interpret the predictions, the scientific conclusion derived based on them automatically is considerate of the variation among biological samples or the physiology of patients. Another factor impacting the credibility of confidence intervals is an adequate identification of all the sources of uncertainty and a correct characterisation and propagation of those, that indeed affect the prediction outcome. To know which sources should be prioritised for the UQ exercise, a prior sensitivity analysis is recommendable (Eck et al. 2016).

### Suggestions for future work

#### Consideration of epistemic uncertainty

In this publication, although different sources of aleatory and epistemic uncertainty were identified, the described methods were mainly focused on the characterisation and propagation of two sources of variability. The protocol integrated only principles of the frequentist approach to probability. Indeed, when quantifying only variability reflecting the natural variability and randomness, the selection of normal distribution with standard deviation as the representation of sample spread was a reasonable decision. This is because real-valued random variables whose distributions are undefined are usually represented using normal distributions. As stated in the Central Limit Theorem, under some conditions, when a large series of random numbers are sampled from any population with a defined mean and sd, the initial distribution converges to a normal distribution as the number of samples increases (Devore and Berk 2012).

However, epistemic uncertainty, also identified in this work, should not be expressed nor modelled using frequentist methods. Instead, the correct way to assess epistemic problems involves the application of the subjective probability theory, the most common application of which is the Bayesian theorem (van de Schoot et al. 2021). This involves starting with an initial belief, known as the prior probability, and updating it when new information becomes available yielding the posterior distribution. Nevertheless, applying Bayesian statistics to estimate the impact of purely epistemic factors (shown in yellow in Fig. 2) on the APD_90_ predictions would require major modifications of the developed uncertainty quantification protocol. But particularly important for this work and more feasible to implement would be the consideration of epistemic uncertainty about the aleatory uncertainties summarised as variability types. This would lead to a quantitative expression of the level of unknown in the metrics defined to characterise specific variability types, for instance, the constant sd value of 0.5 that was assumed to characterise experimental variability. Degrees of belief about the true parameters for this quantity could be derived using either objective measurements or subjective expert judgements. To propagate the uncertainty about variability in the quantity of interest, sampling of the resulting prior distributions could be integrated as part of a two-dimensional Monte Carlo simulation. A result of such a simulation would not be a single distribution of values, but multiple distributions representing the uncertainty about variability (Benford et al. 2018a, b). Coming back to the previous example, the uncertainty about the level of experimental variability would be expressed as several distributions, each of which with a different centre (median or mean) and measure of spread.

### Computational model inputs

There is a high interest in transforming the mixed-platform pre-clinical cardiac safety assessment of novel pharmaceutical products into purely in silico-based methods without the need for extensive experimental testing. Therefore, the structure of our multi-level cardiotoxicity models allows both, experimental as well as predicted inputs. Since computational models, such as the PBPK or QSAR models, are built using experimental data, experimental variability, which was extensively described in this work, is also retained in the training series used for building these models. However, when the plasma drug concentration or the channel-specific IC_50_ are generated computationally, the level of epistemic uncertainty increases due to further limitations in the training data coverage or a high level of subjectivity impacting the parametrisation of the respective source models that predicts them.

For instance, if the intention is to predict proarrhythmic properties of a compound available in a public domain, such as the ChEMBL database (Gaulton et al. 2012), multiples datapoints would be available for the same compound, each of which is produced in a separate experiment following a specific protocol. These data points would first need to be extensively filtered to select the experimental parameters of interest and aggregated using statistical measures such as a mean or the median. This process, together with multiple unconsidered originating from differences in laboratory conditions, experimental design, and other factors, would contribute to the level of epistemic uncertainty. Despite of these factors, the predictive performance of purely computational proarrhythmia prediction systems highly depends on the selected biomarker. As shown by (Beattie et al. 2013), the use of QSAR-derived data to simulate QT-interval shortening may yield nearly as good predictions as those produced using experimental data inputs. Conversely, utilising QSAR data to predict QT-interval prolongation significantly worsens the predictive performance. These two examples show the importance of comprehensive definition of the endpoint being modelled which should always precede the process of uncertainty analysis to ensure a correct determination of model limitations, variability sources, and epistemic factors.

#### QSAR

The most widely accepted standard method for the quantification of reliability and uncertainty associated with QSAR model predictions are methods based on the concept of applicability domain (AD) (Sahlin et al. 2014). Predictions generated for compounds having structurally or physio-chemically similar counterparts in the training set are generally considered reliable. Standard AD methods can be complemented by placing the model within a framework that can estimate the uncertainty levels in every single prediction. An example is the conformal prediction framework which guarantees the maximum allowed frequency of errors which will be committed by the conformal predictor (Alvarsson et al. 2021; Norinder et al. 2014; Svensson et al. 2018). Uncertainty resulting from lack of knowledge (e.g., insufficient training data or anomalous samples in test data), that is predominant in model predictions, is most commonly addressed by applying Bayesian inference, shortly introduced above (Sahlin 2015).

#### PBPK

The arrhythmogenic potential of drug candidates is typically assessed at early stages of drug development when the compound can still be removed from the development pipeline without much economic harm. At these stages, the therapeutic concentration and other PK parameters required to compute the EFTPC are still unknown, but the use of currently described methodologies to estimate point-of-departure concentrations is an interesting approach. These could be compared with the experimental results produced at pre-clinical stages using physiologically based pharmacokinetic (PBPK) modelling to obtain plasma concentrations from the administered doses. PBPK models are mathematical algorithms based on ordinal differential equations (ODEs) describing physiological processes involved in the absorption, distribution, metabolism, and excretion of the drug (Piñero et al. 2018). Variability and uncertainty quantification in PBPK models is often initiated by a parametric sensitivity analysis to identify the PK parameters that are most susceptible. Since PK parameters are subject to inter-individual differences and PK simulations are often liable to lack of full information about the constants and parameters in the ODEs, the UQ methods require combining the frequentist and conditional probabilistic approaches (Kuepfer et al. 2016). Consideration of uncertainty in PBPK simulations would allow to explore a range of clinically relevant drug concentrations, especially at the site of the pharmacological or toxicological action of the drug (e.g., drug-binding site at the ion channel protein in the membrane of ventricular myocytes) (Li et al. 2019a, b).

## Conclusions

In this study, we developed and tested methods for the quantification of the impact of selected variability types on the uncertainty of APD_90_ predictions generated by an in silico multi-level proarrhythmia model. The aim was first to explore the effects of different types of variability, separately and in combination, by quantitatively characterising and propagating them throughout our complex model, and second to replace point predictions with value ranges that can be computed for predefined credibility levels (e.g., 80%) and interpreted as confidence intervals.

The propagation of “experimental variability”, associated with the input IC_50_ values, yielded distributions whose characteristics were defined by the location of the *IV*s within the hyperplane-like structure of model training data. This contrasts with the distributions resulting from the propagation of “inter-individual variability”, linked with the parameters specified in the AP simulation models, whose shape and width were a direct consequence of the methodological assumptions and the predicted spread parameters, respectively. After a simultaneous propagation of both types, the distributions showed a combined effect of both, the non-linear relationship between the *IV*s and APD_90_ and the assumption of normality applied to model outputs. Importantly, combining two sources of variability did not lead to additive results, meaning that the combined result is not their sum.

Further, we showed how such distributions can be used to compute the proarrhythmia biomarker predictions together with value intervals of certain credibility. One of the main conclusions arising from this analysis was that the actual biomarker prediction remains nearly unchanged when the simulations are performed, as compared to the initial method without UQ. Although we do not claim the undoubtful accuracy of these results, we consider that such representation of the predictions has excellent advantages over single-point estimates. These mainly include the possibility to inspect values that would be produced in experiments or for individuals that do not represent the exact centre of the distribution from which they were drawn. Hence, it allows to protect individuals who are more prone to develop cardiac arrhythmias or TdP, since interval ranges may cross the boundaries of different risk classes. Moreover, they provide a more realistic view on predictions in the context of drug candidate prioritisation and validation of clinical results, since the presence of uncertainty resulting from variability is usually neglected at these assessment stages.

## Supplementary Information

Below is the link to the electronic supplementary material.

Supplementary file1 (DOCX 22 KB)

## Data Availability

Data are provided in the github: https://github.com/phi-grib/Cardiotox_uncertainty
