# Supplementary material for: Uncertainty assessment of proarrhythmia predictions derived from multi-level in silico models
Source: Arch Toxicol. 2023 Aug 1;97(10):2721–40. doi: 10.1007/s00204-023-03557-6 (PMC10474996; doi:10.1007/s00204-023-03557-6)
Supplement: Supplementary file 1 — Supplementary file1 (DOCX 22 KB) [file 204_2023_3557_MOESM1_ESM.docx]

Supplementary materials

**Uncertainty assessment of proarrhythmia predictions derived from multi-level *in silico* models**

Karolina Kopańska^1*^, Pablo Rodríguez-Belenguer^1,2*^, Jordi Llopis^3^, Beatriz Trénor^3^, Javier Saiz^3^, Manuel Pastor^1^

*^1^Research Programme on Biomedical Informatics (GRIB), Department of Medicine and Life Sciences, Universitat Pompeu Fabra, Hospital del Mar Medical Research Institute. Barcelona, Spain*

*^2^Department of Pharmacy and Pharmaceutical Technology and Parasitology, Universitat de València, Valencia, Spain*

*^3^Centro de Investigación e Innovación en Bioingeniería (Ci2B), Universitat Politècnica de València, Valencia, Spain*

*^*^Both authors have contributed equally*

# Detailed Results

**Table 1:** Predicted APD_90_, ranges of value intervals (CI 80%) and their corresponding computed for 12 CiPA compounds considering. Simulation A: Experimental variability, Simulation B: Inter-individual variability and Simulation C: Combination of experimental and inter-individual variability.

**Simulation A**

|  | APD_90_ | CI_80%_ | CI_width_ |
| --- | --- | --- | --- |
| Bepridil | 296.1 | [271.6-364.5] | 92.9 |
| Dofetilide | 268.4 | [264.8-281.0] | 16.3 |
| Quinidine | 445.9 | [338.8-555.5] | 216.6 |
| Sotalol | 271.6 | [266.0-295.7] | 29.7 |
| Chlorpromazine | 272.5 | [266.3-296.9] | 30.6 |
| Cisapride | 269.6 | [265.3-286.9] | 21.6 |
| Ondansetron | 277.8 | [266.0-321.2] | 55.2 |
| Terfenadine | 267.7 | [264.5-278.6] | 14.0 |
| Diltiazem | 265.0 | [264.1-268.4] | 4.3 |
| Mexiletine | 256.2 | [254.3-267.2] | 12.9 |
| Ranolazine | 286.1 | [269.6-342.8] | 73.2 |
| Verapamil | 286.9 | [269.4-340.1] | 70.7 |

**Simulation B**

|  | APD_90_ | CI_80%_ | CI_width_ |
| --- | --- | --- | --- |
| Bepridil | 295.2 | [253.8-341.3] | 87.4 |
| Dofetilide | 269.1 | [226.7-310.4] | 83.6 |
| Quinidine | 445.9 | [400.4-491.9] | 91.4 |
| Sotalol | 271.0 | [224.5-317.4] | 92.9 |
| Chlorpromazine | 272.4 | [228.5-317.3] | 88.8 |
| Cisapride | 267.4 | [225.9-310.4] | 84.6 |
| Ondansetron | 276.1 | [231.3-322.2] | 90.9 |
| Terfenadine | 265.4 | [222.2-309.2] | 87.0 |
| Diltiazem | 265.7 | [224.5-307.8] | 83.3 |
| Mexiletine | 254.0 | [214.4-293.7] | 79.3 |
| Ranolazine | 286.6 | [244.4-329.0] | 84.6 |
| Verapamil | 287.6 | [242.6-329.8] | 87.2 |

**Simulation C**

|  | APD_90_ | CI_80%_ | CI_width_ |
| --- | --- | --- | --- |
| Bepridil | 300.8 | [247.4-376.1] | 128.7 |
| Dofetilide | 271.3 | [228.8-315.5] | 86.8 |
| Quinidine | 442.3 | [335.3-565.0] | 229.7 |
| Sotalol | 275.8 | [228.1-324.2] | 96.0 |
| Chlorpromazine | 276.3 | [229.1-327.5] | 98.4 |
| Cisapride | 271.8 | [226.6-320.7] | 94.1 |
| Ondansetron | 282.8 | [230.8-343.7] | 112.9 |
| Terfenadine | 267.1 | [224.1-312.2] | 88.0 |
| Diltiazem | 266.8 | [224.5-309.3] | 84.8 |
| Mexiletine | 258.8 | [217.9-301.0] | 83.1 |
| Ranolazine | 292.9 | [242.4-359.2] | 116.8 |
| Verapamil | 292.8 | [241.8-355.2] | 113.4 |
